# Baseline neurofilament light chain and brain-derived neurotrophic factor levels predict development of aggressive multiple sclerosis

**DOI:** 10.55730/1300-0144.6067

**Published:** 2025-07-13

**Authors:** Ruziye EROL YILDIZ, Ece AKBAYIR, Tuğçe KIZILAY, Ayça Simay ERSÖZ, Duygu ÖZKAN YAŞARGÜN, Devran SÜER, Vuslat YILMAZ, Erdem TÜZÜN, Recai TÜRKOĞLU

**Affiliations:** 1 Department of Neurology, University of Health Sciences, Haydarpaşa Numune Training and Research Hospital, İstanbul, Turkiye; 2 Department of Neuroscience, Aziz Sancar Institute of Experimental Medicine, İstanbul University, İstanbul, Turkiye; 3 Department of Language and Speech Therapy, Faculty of Health Sciences, İstanbul Atlas University, İstanbul, Turkiye

**Keywords:** Multiple sclerosis, brain-derived neurotrophic factor, neurofilament light chain, disability, autoimmunity

## Abstract

**Background/aim:**

Patients with multiple sclerosis (MS) may present with a rapidly disabling clinical course in the first few years of disease. It is imperative to find biomarkers to predict patients with aggressive MS (AMS) and have an opportunity to prevent disability accumulation through appropriate treatment strategies. Our aim was to explore the prognostic value of neurofilament light chain (NFL) and brain-derived neurotrophic factor (BDNF) levels in cerebrospinal fluid (CSF) obtained in the early stages of MS.

**Materials and methods:**

Relapsing-remitting multiple sclerosis (RRMS) patients presenting with first-time attacks were screened, of which 26 fulfilled AMS criteria in the 3-year follow-up period. In addition, 27 age/sex-matched RRMS patients without AMS (non-AMS) were included. Baseline NFL and BDNF levels were measured in CSF obtained during the remission period following the first MS attack. Disease activity was monitored for 3 years by periodic expanded disability status scale (EDSS), cranial-spinal MRI assessments, and no evidence of disease activity (NEDA)-3 was determined.

**Results:**

AMS patients showed significantly higher attack numbers, EDSS scores, MRI lesions, and baseline NFL levels compared to non-AMS patients. Baseline BDNF levels were significantly lower than in non-AMS patients. NFL/BDNF levels were correlated with number of attacks and/or EDSS scores at the third year follow-up. Patients with NEDA-3 showed significantly lower baseline NFL and higher BDNF levels than those without NEDA-3. Receiver operating characteristic curve analysis showed the highest specificity for CSF BDNF measurements in predicting AMS conversion.

**Conclusion:**

Baseline NFL and BDNF levels effectively predict the development of AMS emerging early in the course of MS. Combined use of these molecular markers with MRI results may enable early diagnosis and appropriate therapeutic intervention of AMS.

## 1. Introduction

Multiple sclerosis (MS), the most common autoimmune demyelinating disease of the brain, may present with rapidly increasing numbers of attacks, MRI lesions, and failure of complete symptom resolution under immunomodulating drug treatment. This so-called highly active or aggressive MS (AMS) may lead to early accumulation of disability and high expanded disability status scale (EDSS) scores over the first few years of disease [1].

It is vital to recognize and predict the development of AMS as early as possible to prevent disability accretion. Thus, early-stage biomarkers are required. The aggressive clinical course of MS has been associated with cigarette smoking, frequent attacks, partial treatment response to attacks, and high T2/contrast enhancing MRI lesion load [1]. Although high MRI lesion load (e.g., >20 T2 lesions in first-attack MRI) has often been linked to rapidly progressive MS [2], the sensitivity and specificity of this finding are 85% and 76%, respectively. Therefore, many MS patients not displaying aggressive disease features may also present with increased number of MRI lesions and vice versa.

Neurofilament light chain (NFL) is an abundant cytoskeletal protein exclusively expressed in neurons and is a promising predictive biomarker of disability accumulation in MS. NFL is associated with MS relapsing activity and may have prognostic utility in predicting relapse-related disease progression and brain damage [3]. Increased baseline levels of NFL have been associated with the emergence of AMS [4]. Also, NFL levels are significantly reduced in patients with AMS following autologous hematopoietic stem cell transplantation in parallel with amelioration of clinical and neuroimaging features of the disease [5].

Brain-derived neurotrophic factor (BDNF) is a neurotrophin mostly produced by glial cells and is involved in tissue regeneration and neurodegenerative disorders [6,7]. More recently, BDNF has been associated with disease outcome in MS. BDNF levels are lower in relapsing-remitting MS (RRMS) patients than in healthy controls [8], and levels of BDNF correlate with EDSS scores of RRMS patients [9,10]. Notably, the association between BDNF and disability progression in AMS has not been previously investigated.

In this study, the prognostic ability of NFL and BDNF as baseline markers of disease severity was examined in AMS patients. Thus, we explored the possible associations between cerebrospinal fluid (CSF), NFL/BDNF levels, and clinical features of MS to assess the potential biomarker value of these molecules in predicting and monitoring AMS development. We comparatively investigated the biomarker utility of 2 potential biomarkers of MS acting through diverse biological mechanisms: NFL, a well-established marker of neuro-axonal loss and unfavorable prognosis in MS [4], and BDNF, a neuroprotective mediator also representing antiinflammatory glial activity [11].

## 2. Materials and methods

### 2.1. Subjects

In this retrospective, exploratory, observational study, we examined RRMS patients with available baseline CSF samples and prospective follow-up in our MS unit. Out of 327 RRMS patients (as per the revised McDonald criteria [12]) admitted to our outpatient MS clinic with their first clinical event between January 2019–January 2020, 33 patients fulfilled the AMS criteria [13] during the 3-year follow-up period. Several criteria that are used to define AMS require a follow-up period of more than 10 years and development of EDSS ≥6.0 [2,14,15]. However, at this disability level, walking aid is required and daily life activities are significantly affected. Since it is difficult to revert disability at this stage of MS and our aim was to determine a biomarker that identifies patients at high risk of AMS development in the early stages of disease, we used the criteria of Rush et al. [13] that mandates at least one of the following indications: 1) confirmed EDSS ≥4.0 within 5 years of disease onset, 2) ≥2 relapses with incomplete recovery within 12 months, 3) ≥2 MRI with new or enlarging T2 or contrast enhancing lesions within 12 months of immunomodulating drug treatment, 4) no treatment effect during the first year of immunomodulating drug treatment.

In our outpatient clinic, CSF samples were collected and archived from the 33 RRMS patients presenting with AMS. Seven AMS patients were excluded since their CSF samples were obtained within 3 months of the onset of the attack. The status of the attack may cause extreme alterations in CSF levels of NFL [16,17]. This left 26 AMS patients. CSF samples from 27 age/sex-matched non-AMS RRMS patients, who were admitted during the same period of time, were also included for comparison. CSF samples had been obtained from all participants during the remission period between 107–120 days following the first clinical MS attack. During CSF sampling, none of the included RRMS patients were undergoing treatment with immunosuppressive or immunomodulating agents. Neither had they developed another MS attack after the first one, had coexisting autoimmune diseases, cardiovascular conditions, history of malignancy, pregnancy, nor clinically active infections.

Patients were evaluated every 6 months for 3 years and whenever they had attacks through neurological examination, contrast-enhanced cranial and spinal MRI (with the same 1.5 T MRI device), and EDSS assessments. Timed 25-foot walk and 9-hole peg tests were performed during CSF baseline sampling and in the third year of follow-up. No evidence of disease activity (NEDA)-3 was determined based on the absence of clinical relapses, MRI evidence of disease activity, and disability worsening in the follow-up period.

The study protocol was approved by the Institutional Review Board (HNEAH-KAEK 2019/116). The study was conducted in accordance with the ethical principles of the Declaration of Helsinki. All subjects provided written informed consent prior to any study related procedure.

### 2.2. ELISA

All CSF samples obtained during the remission period following the first clinical MS attack were aliquoted and stored at −80 °C until use. CSF NFL and BDNF levels were measured with ELISA kits (Uman Diagnostics, Umea, Sweden, and Elabscience, Houston, TX, USA, respectively) according to the manufacturer’s instructions. Optical densities were measured at 450 nm and concentrations were calculated by reference to standard curves. The results were expressed as pg/mL.

### 2.3. Statistical analysis

Pairwise comparisons were performed with Mann–Whitney U, chi-square, and unpaired t-tests, as required. Correlation analyses were performed with Pearson or Spearman correlation tests. Sensitivity and specificity for AMS development were calculated using receiver operating characteristic (ROC) curve analysis and thresholds were selected using the Youden index. A p-value of <0.05 was considered as statistically significant.

## 3. Results

### 3.1. Clinical features and follow-up results of RRMS patients

Among 26 AMS patients, the Rush et al. [13] criteria were satisfied. EDSS scores ≥4.0 within 5 years of onset were found in 10 patients. At least 2 or more relapses with incomplete resolution in the past year were experienced by 19 patients. Sixteen patients had more than 2 MRIs showing new or enlarging T2 lesions or gadolinium-enhancing lesions despite treatment. One or more immunomodulating treatments had no apparent effect for up to 1 year in 13 patients.

AMS and non-AMS patients had comparable age and sex at MS onset (Table 1). The total and annual number of attacks was significantly higher in AMS patients during the 3-year follow-up period. While the baseline EDSS scores were comparable, AMS patients had significantly higher EDSS scores than non-AMS patients after 1 and 3 years. EDSS scores of at least 3 were found in 16 out of 26 AMS patients, whereas all of the non-AMS patients had EDSS scores below 3 during the follow-up period. Timed 25-foot walk and 9-hole peg tests were more impaired in AMS patients at baseline and at 3 years. Significantly higher number of AMS patients had more than 20 MRI lesions during the first attack. During the follow-up, AMS patients developed a significantly higher number of supratentorial lesions, whereas their infratentorial lesions were comparable with non-AMS patients. Although AMS patients had a lower prevalence of NEDA-3 at the first and third year of the follow-up, this difference became statistically significant at the third year clinical assessment (Table 1).

### 3.2. CSF NFL and BDNF levels and association between clinical follow-up features

**Figure 1:**
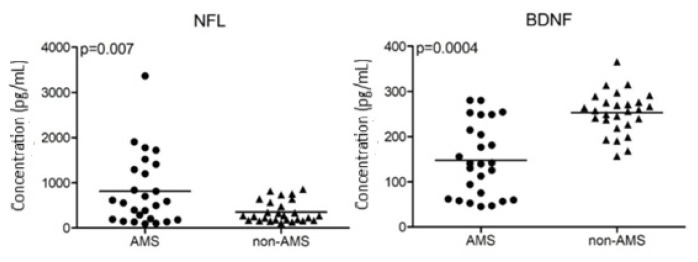
Comparison of baseline CSF NFL (left) and BDNF (right) levels of RRMS patients with aggressive (AMS) and nonaggressive (non-AMS) disease courses. Horizontal lines indicate mean values. Statistical analysis was performed with paired Student’s t-test and p-values are shown in the upper left corners of the panels.

At baseline, AMS patients showed significantly higher CSF NFL and significantly lower CSF BDNF levels compared to non-AMS patients. All of the non-AMS patients had CSF NFL levels <1000 pg/mL, whereas 8 out of 26 AMS patients had levels >1000 pg/mL. CSF BDNF levels were >150 pg/mL in all non-AMS patients, whereas 15 out of 26 AMS patients had levels <150 pg/mL (Figure 1). Only 4 AMS patients had both >1000 pg/mL CSF NFL and <150 pg/mL CSF BDNF. There was no significant correlation between CSF BDNF and NFL levels (p = 0.625, R = −0.072).

CSF NFL levels were only significantly correlated with the number of attacks during the follow-up period (p < 0.001, R = 0.483), whereas CSF BDNF levels were significantly correlated with both number of attacks during the follow-up period (p = 0.031, R = −0.309) and EDSS score at the third year of the follow-up (p = 0.043, R = −0.300). There was no significant correlation between NFL or BDNF levels versus other continuous parameters listed in Table 1. Of the 8 patients with CSF NFL >1000 pg/mL, 5 had >20 T2 MRI lesions during the first attack. Of the 15 patients with <150 pg/mL CSF BDNF, 8 had >20 T2 MRI lesions during the first attack. There was no significant difference between baseline CSF NFL of RRMS patients with and without NEDA-3 at the first year of follow-up (444.3 ± 362.4 pg/mL and 718.4 ± 752.2 pg/mL, respectively, p = 0.098). The same was true for CSF BDNF levels (244.7 ± 47.3 pg/mL and 212.4 ± 116.1 pg/mL, respectively, p = 0.108). Conversely, at the third year of follow-up, RRMS patients with NEDA-3 showed significantly lower baseline NFL compared to those without (328.9 ± 208.0 pg/mL and 702.4 ± 696.8 pg/mL, respectively, p = 0.003) and significantly higher baseline BDNF (251.1 ± 34.0 pg/mL and 216.0 ± 108.0 pg/mL, respectively, p = 0.044).

**Figure 2:**
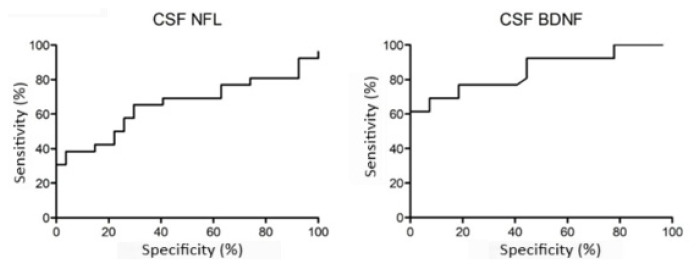
ROC curves for NFL and BDNF concentrations in CSF samples of non-AMS (RRMS patients who did not convert to aggressive MS during follow-up) and AMS (RRMS patients who converted to aggressive MS during follow-up) patients, where the binary outcome is defined as AMS vs. non-AMS.

**Figure 3:**
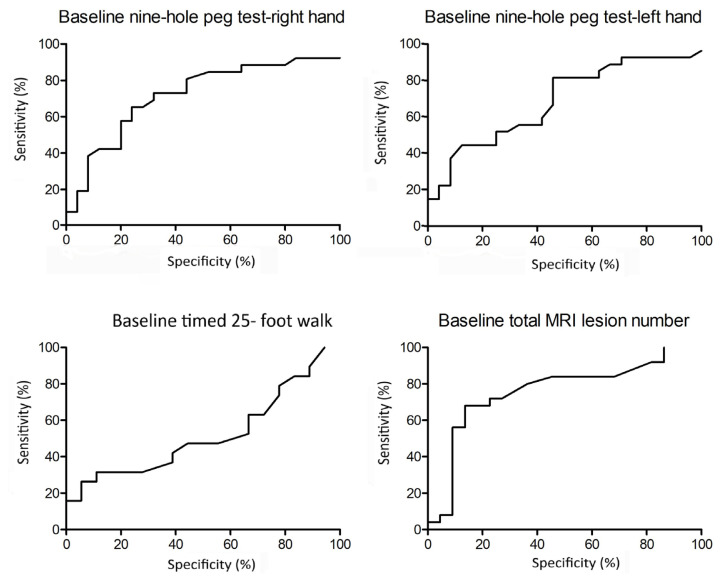
ROC curves for baseline 9-hole peg test, timed 25-foot walk test, and total MRI lesion number of non-AMS (RRMS patients who did not convert to aggressive MS during follow-up) and AMS (RRMS patients who converted to aggressive MS during follow-up) patients, where the binary outcome is defined as AMS vs. non-AMS.

The diagnostic sensitivity, specificity, and threshold values of CSF levels of NFL and BDNF were calculated using ROC curves and the Youden index (Figure 2). The best area under curve (AUC) value was for CSF BDNF level measurements at a threshold of 156.4 pg/mL. Although CSF NFL and BDNF level measurements yielded comparable sensitivity values at thresholds of 362.7 pg/mL (65.4%) and 156.4 pg/mL (61.5%), respectively, the specificity of CSF BDNF in predicting AMS conversion was far higher (100%) than that of the CSF NFL level (70.4%) (Table 2). ROC curves were also constructed for baseline timed 25-foot walk and 9-hole peg tests and total baseline MRI lesion load (number of supratentorial, infratentorial and spinal lesions at baseline MRI) (Figure 3). While the 25-foot walk test did not yield a significant result, 9-hole peg tests for right and left hands and MRI lesion load analysis yielded p-values between 0.002 and 0.024, sensitivity values between 26.3% and 65.4%, and specificity values between 76.0% and 87.5% (Table 2). CSF BDNF level had the highest sensitivity, likelihood ratio, Youden index, and AUC as well as the lowest p-values among all parameters analyzed by ROC curves (Table 2).

## 4. Discussion

In this study, baseline CSF NFL levels of AMS patients were higher than those of non-AMS patients, as previously shown [4]. Baseline CSF BDNF levels of AMS patients were also significantly lower than those of non-AMS patients, showed weak correlation with third year EDSS and total attack numbers in a 3-year follow-up period, and were associated with NEDA-3. To the best of our knowledge, our results show an association between low CSF BDNF levels and high MS disease activity for the first time. It is particularly noteworthy that patients with baseline CSF levels of NFL <1000 pg/mL and BDNF >150 pg/mL were less likely to develop an aggressive MS course. A BDNF level of <150 pg/mL predicted a non-AMS disease course with 100% specificity, lending further support to the prognostic biomarker value of BDNF for AMS development in the early stages of MS. Furthermore, baseline MRI lesion, 9-hole peg test, and timed 25-foot walk test parameters were not superior to baseline BDNF level measurements. Confirmation of these results in larger MS cohorts is thus warranted.

Among several published criteria for determining AMS, we selected one that requires a relatively shorter follow-up period and lower EDSS scores [13]. AMS patients in our study showed significantly increased disease activity in terms of number of attacks, MRI lesion numbers, EDSS progression, and lower NEDA-3 prevalence, justifying utilization of these criteria. Also, a roughly 10% AMS prevalence among the first-attack RRMS patients falls within the previously reported range of AMS prevalence in an MS cohort [1]. The association between AMS development and a higher prevalence of the first MRI showing more than 20 T2 lesions [2] is also verified by our study. Our results may also suggest that baseline CSF assessment of NFL/BDNF levels and baseline MRI lesion load may be more helpful than baseline EDSS evaluation in predicting high disease activity in MS.

Notably, even at baseline assessment before fulfilling the criteria for AMS, 9-hole peg and timed 25-foot walk tests were significantly worse in AMS patients compared to non-AMS patients. This finding indicates that affliction of the central nervous system and onset of neuroaxonal loss starts at the very early stages of MS and possibly before the first attack, especially for AMS patients. Another notable finding was that, in AMS, the accrual of clinical progression was more closely associated with an increase of cerebral rather than spinal lesions.

NFL is a marker of neuroaxonal loss. This means that increased CSF levels of NFL are associated with a more severe disease course and increased disability whether MS patients develop an aggressive disease course or not [3–5]. Cross-sectional NFL levels do not necessarily correlate with EDSS scores (as was the case in our study) and persistently elevated levels of NFL determined at multiple time points are more closely associated with disability [18,19]. However, NFL levels were associated with NEDA-3 occurrence, as reported previously [20–22].

RRMS patients have significantly lower BDNF levels than healthy controls [8] and BDNF levels of RRMS patients show significant correlation with EDSS scores as shown previously [9,10] and in this study. Further reduction of BDNF appears to be linked to conversion from RRMS to secondary progressive MS [10,23]. The mechanisms by which reduced BDNF contribute to disease activity in MS are less well understood. Notably, CSF BDNF levels did not show an association with CSF NFL levels and MRI lesion load at baseline or during follow-up, suggesting that BDNF is linked to disease activity of MS through mechanisms that do not necessarily involve neuroaxonal loss or development of inflammatory brain lesions. BDNF is released by microglia and reactive astrocytes and acts as a neuroprotective agent [24,25]. Increased levels of glial fibrillary acidic protein (GFAP), a marker of astrocytic activity, have been associated with a more aggressive disease course in MS [26,27]. Glial BDNF production is considered to be decreased following the conversion of glial cells to proinflammatory phenotypes [11,28]. As shown in rodent studies, BDNF is required for induction of oligodendrocyte lineage cells, production of myelin proteins, and stimulation of axonal outgrowth [29,30]. It is thus tempting to speculate that reduction of BDNF levels after the first MS attack may be an indicator of early activation of proinflammatory glial cells in widespread brain regions, thereby contributing to high disease activity through the release of mediators that are toxic for neurons and oligodendrocytes.

A limitation of our study was a low number of patients due to the strict and rigorous inclusion criteria. It was also notable that 10 out of 26 AMS patients did not attain an EDSS score of 3. This might be explained by short follow-up time and differential effects of diverse immunomodulating drug combinations. Due to a high variety of drug combinations used in our study, the low number of patients, and low statistical power, treatment type could not be included as a variable in the statistical analysis. Therefore, confirmation of these results in larger MS cohorts receiving different treatment types is warranted. In conclusion, our pilot exploratory study showed for the first time that BDNF may have utility as a predictive biomarker for AMS, comparable to NFL. Increased BDNF/NFL levels and MRI lesion load after the first attack appear to be predictive for induction of AMS, albeit through independent mechanisms. Combined use of these 3 clinical features may lead to more precise monitoring and determination of an aggressive disease course in MS.

## Figures and Tables

**Table 1:** Clinical features of aggressive (AMS) and nonaggressive (non-AMS) RRMS patients.

	AMS (n=26)	non-AMS (n=27)	p-value
**Age**	32.8 ± 9.4	33.2 ± 9.2	0.314
**Sex (female/male)**	17/9	17/10	0.854
**Age at MS onset**	31.2 ± 9.1	31.0 ± 8.9	0.269
**Total number of attacks during follow-up**	3.3 ± 1.3	2.3 ± 1.0	**<0.001**
**Annual number of attacks during follow-up**	3.1 ± 1.1	1.9 ± 0.8	**<0.001**
**EDSS (baseline)**	2.3 ± 0.7	1.9 ± 0.6	0.071
**EDSS (1st year)**	2.8 ± 1.1	2.0 ± 0.6	**0.004**
**EDSS (3rd year)**	3.3 ± 1.3	1.9 ± 0.7	**<0.001**
**Number of patients with EDSS ≥ 3.0 during follow-up**	16	0	**<0.001**
**Time to EDSS 3.0 (years)**	1.0 ± 1.3	NA	NA
**Baseline nine-hole peg test (right)**	25.6 ± 7.8	21.2 ± 4.8	**0.005**
**Baseline nine-hole peg test (left)**	25.3 ± 7.2	21.4 ± 3.7	**0.008**
**3rd-year nine-hole peg test (right)**	28.2 ± 8.5	22.0 ±4.2	**0.002**
**3rd-year nine-hole peg test (left)**	31.0 ± 5.2	22.2. ± 4.4	**0.007**
**Baseline timed 25-foot walk**	8.5 ± 3.9	7.1 ± 1.5	**0.036**
**3rd-year timed 25-foot walk**	9.7 ± 4.5	7.7 ± 1.6	**0.021**
**Number of patients with >20 MR lesions during the first attack**	14	4	**0.003**
**Total number of ST cerebral lesions during follow-up**	28.7 ± 14.6	15.9 ± 15.1	**0.038**
**Total number of IT cerebral lesions during follow-up**	2.6 ± 2.9	0.6 ± 0.8	0.066
**Total number of spinal lesions during follow-up**	1.7 ± 1.1	1.0 ± 1.0	0.103
**Patients with NEDA-3 (first year)**	8	15	0.069
**Patients with NEDA-3 (third year)**	0	12	**<0.001**

EDSS, expanded disability status scale; ST, supratentorial; IF, infratentorial; NEDA-3, no evidence of disease activity (absence of clinical relapses, MRI evidence of disease activity and disability worsening) during the follow-up period; NA, not applicable.

Parametric variables are depicted as mean ± standard deviation. All continuous parametric variables were compared using unpaired Student’s t-test, categorical variables were compared using chi-square test, and disability scores were compared using the Mann–Whitney U test. Significant p-values are denoted by bold characters.

**Table 2:** Sensitivity and specificity values of CSF NFL and BDNF levels for thresholds determined by respective Youden indexes.

	Cut-off value	Sensitivity%	95% CI	Specificity%	95% CI	LR	Youden index	AUC, p-value
**CSF NFL (pg/mL)**	>362.7	65.4	44.3% to 82.8%	70.4	49.8% to 86.2%	2.2	0.357	0.655, 0.042
**CSF BDNF (pg/mL)**	<156.4	61.5	40.6% to 79.8%	100.0	87.2% to 100.0%	9.3	0.615	0.853, <0.001
**Nine-hole peg test-right hand (seconds)**	>22.9	65.4	44.3% to 76.0%	76.0	54.9% to 90.6%	2.7	0.414	0.718, 0.008
**Nine-hole peg test-left hand (seconds)**	>24.9	44.4	25.5% to 64.7%	87.5	67.6% to 97.3%	3.6	0.319	0.684, 0.024
**Timed 25-foot walk (seconds)**	>9.6	26.3	9.1% to 51.2%	94.4	72.7% to 99.9%	4.7	0.208	0.520, 0.832
**Total MRI lesion numbe**r	>18.5	68.0	46.5% to 85.0%	86.4	65.1% to 97.1%	4.9	0.544	0.758, 0.002

CSF, cerebrospinal fluid; CI, confidence interval; LR, likelihood ratio; AUC, area under the curve.
